# Clinical Study of Restless Leg Syndrome Accompanied by Psychological Symptoms Induced by High-Dose Treatment With Madopar

**DOI:** 10.3389/fpsyt.2019.00360

**Published:** 2019-05-24

**Authors:** Lei Zhu, Jing Li, Chongyang Ren, Mei Zhang, Min Xue, Chuanqing Yu, Weili Zhang

**Affiliations:** ^1^Department of Neurology, The First People’s Hospital of Huainan, The First Affiliated Hospital of Anhui University of Science and Technology, Huainan, China; ^2^State Key Laboratory of Cardiovascular Disease, Fuwai Hospital, National Center for Cardiovascular Diseases, Chinese Academy of Medical Sciences and Peking Union Medical College, Beijing, China; ^3^Beijing Institute for Brain Disorders, Center for Brain Disorders Research, Capital Medical University, Beijing, China

**Keywords:** Madopar, restless leg syndrome, anxiety, depression, psychological symptoms

## Abstract

**Objectives:** Some neurological disorders demonstrate indistinguishable psychological symptoms at an early stage, especially when accompanied by jitters similar to those in Parkinson’s disease. During dopamine replacement therapy, some patients display restless leg syndrome (RLS)-like symptoms. Therefore, we aimed to analyze treatment strategies and the prognosis of RLS caused by high-dose Madopar.

**Methods:** Nine patients who were misdiagnosed with Parkinson’s disease, taking a high dose of Madopar, and showed symptoms of anxiety, depression, and somatization were recruited. Clinical data were collected, and strategies of treatment and prognosis were analyzed.

**Results:** Seven patients demonstrated varying degrees of anxiety and depression, and the other two cases were misdiagnosed as Parkinson’s disease. During Madopar treatment, patients gradually showed aggravated symptoms, including swelling, numbness, pain, and other sensory abnormalities in both lower extremities, which spread to both upper extremities in a few patients. Among the seven patients, symptoms of anxiety, depression, insomnia, and somatization significantly worsened during the observation period. The average time from taking Madopar to the appearance of RLS was 2.6 ± 0.6 months, the average time to clinical diagnosis was 18.17 ± 9.40 months, and the average dosage of Madopar was 1.44 ± 0.21 g per day. Gradually reducing the Madopar dosage and administering a small dose of long-acting dopamine preparation greatly alleviated the symptoms after 3 months.

**Conclusion:** A high dose of Madopar can cause RLS-like symptoms accompanied by anxiety, depression, insomnia, and other mental health symptoms. These symptoms should be more closely monitored by clinicians.

## Introduction

Psychiatric symptoms, such as anxiety, depression, insomnia, and somatization, are the clinical manifestations of common psychiatric and neurological diseases. Many neurological disorders show these indistinguishable psychological symptoms in the early stages, especially when symptoms similar to Parkinson’s disease (e.g., difficulty walking, stiff limbs, and tremors) are present. These patients are likely to be misdiagnosed with Parkinson’s disease and are treated with dopamine replacement therapy; however, in rare cases, increasing the dosage of dopamine can elicit restless leg syndrome (RLS). Extended durations of these psychiatric symptoms can be detrimental to the patient’s physical and mental health.

The present study assessed a group of patients who were misdiagnosed with Parkinson’s disease and were administered large doses of Madopar. All the patients exhibited rare RLS-like symptoms, such as difficulty in walking, stiff limbs, and tremors, which were accompanied by anxiety, depression, and other psychiatric symptoms. Clinical data of all patients were collected, and strategies of treatment and prognosis were analyzed.

## Materials and Methods

### Patients

The present study was approved by the Ethics Committee of the First People’s Hospital of Huainan, and written informed consent was provided. Twelve patients demonstrating symptoms of anxiety, depression, and somatization due to misdiagnosis of Parkinson’s disease and taking a large dose of Madopar were identified and recruited from January 2010 to December 2017. Two Parkinson’s disease patients did not meet the inclusion criteria, and one patient declined to follow up. Therefore, nine patients (47–78 years old) were enrolled. All nine patients were hospitalized, and after a detailed evaluation of medical history, rigorous neurological physical examination, and related auxiliary tests, they were determined to not meet the criteria for Parkinson’s disease according to the British Parkinson’s Disease Society (1). Anxiety, depression, insomnia, and somatization symptoms were diagnosed as depression and anxiety disorders according to the *Diagnostic and Statistical Manual of Mental Disorders, Fifth Edition* (DSM-5) (2). All patients underwent general routine examinations as well as biochemical and imaging examinations. No abnormalities were noted except for the primary disease. The diagnosis for RLS was based on clinical criteria (3) and included an urge to move the legs, usually associated with unpleasant sensations; symptoms occurring during periods of rest, such as sitting or lying down; symptoms relieved by movement; and worsened symptoms in the evening or night.

The education level of all patients was above primary school, and they could independently complete the questionnaire without communication barriers. All patients agreed to follow up.

### Laboratory and Imaging Examinations

Routine blood, urine, fecal, serum glucose level, liver and kidney function, thyroxine, and electrolyte laboratory and physical examinations were conducted. Electroencephalogram and brain magnetic resonance imaging were performed in all patients.

### Clinical Evaluation and Follow-Up

Severity of RLS was evaluated on the basis of the International RLS Rating Scale (IRLS-RS) (4). The diagnosis and severity of insomnia, anxiety, and depression in all patients were assessed by two neurologists and a psychiatrist according to the Insomnia Severity Index (ISI) (5), Hamilton Anxiety Rating Scale (Hamilton) (6), Hamilton Depression Rating Scale (HDRS) (7), and DSM-5 diagnostic criteria (2) combined with clinical symptoms and signs. Follow-up data for all patients with RLS were obtained during face-to-face or telephone interviews.

#### Clinical Research Flow

**Figure 1:**
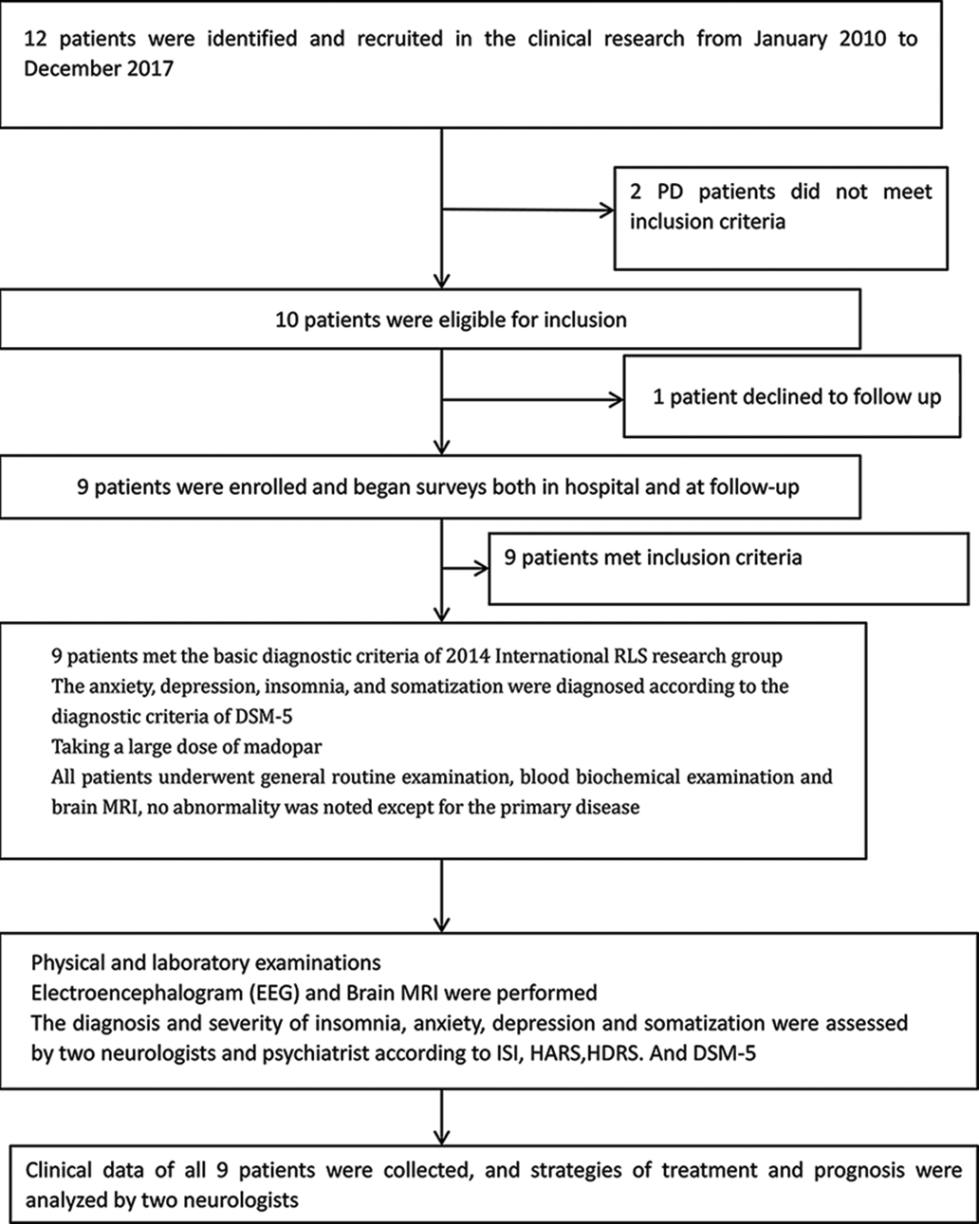
Large doses of Madapor.

The clinical study flow is shown in **Figure 1**.

### Statistical Analysis

All statistical analyses were performed using Statistical Product and Service Solutions (SPSS) version 19.0 (SPSS Inc., Chicago, IL, USA). The normality of the distribution was assessed using the Kolmogorov–Smirnov test. Normally distributed quantitative data were presented as “mean ± standard deviation (SD).” The international RLS scores of patients before and after treatment were compared by *t*-test. The anxiety, depression, and insomnia scores of patients before and after taking a large dose of Madopar were compared using the Student’s *t*-test. *P* values <0.05 were considered significant.

## Results

Nine patients took Madopar orally due to being misdiagnosed with Parkinson’s disease, and the starting dosage ranged from 1/2 to 1 tablet (0.25 g/tablet). All patients gradually increased the amount of medication administered. Some were under the guidance of a doctor, but then to achieve the “curative effect,” patients increased the amount of medication themselves. Some patients increased their doses by themselves from the beginning (i.e., without the doctor’s assistance). The amount of medication in most patients was 2–3 tablets per dose, 3–4 times per day, which was at maximum 5 tablets per dose, 3–5 times a day in one case. When the average dosage reached 6–8 tablets per day and the duration of administration lasted 2–4 weeks, the onset of bilateral lower limb discomfort appeared. Initially, the symptoms were minimal, which did not alert the attention of the patients. As the medication dosage and duration increased, so did the symptoms, which appeared as unexplained abnormal sensations in both lower extremities to varying degrees, such as numbness, swelling, crawling, burning, and traction pain at night. The symptoms could be temporarily reduced by activity, which forced patients to stay out of bed for exercises, which affected their sleep. As a result, patients typically increased the dose of Madopar, which could reduce the symptoms, especially when the symptoms were unbearable. The increasing dosage of Madopar could effectively improve the symptoms, and thus forced patients to increase the amount of medication.

During this cycle, when symptoms appeared during the daytime, the upper limbs and occasionally the entire body displayed varying degrees of involvement. As shown in **Table 1**, the average time from the use of the Madopar to the onset of RLS symptoms was 2.61 ± 0.60 months. For symptoms to appear, the minimum of the average daily dose of Madopar was 1.44 ± 0.21 g; moreover, the average duration for the nine patients with RLS from the time of high-dose Madopar administration to the time of hospital admissions was 18.17 ± 9.40 months. The original symptoms of anxiety, depression, insomnia, and general discomfort worsened in seven patients before onset of the disease. The other two cases displayed anxiety, depression, and insomnia, as well as whole-body burning-like and mobile pain, accompanied by the gradually aggravated discomfort of the bilateral lower limbs. As shown in **Table 2**, the symptoms of anxiety, depression, and insomnia were significantly worse in all nine patients after taking a large dose of Madopar (*P* < 0.0001, *P* < 0.05, *P* < 0.0001).

**Table 1:** Demographic and clinical characteristics of the nine patients.

Case	Gender	Age (years)	Diagnosis	Madopar dosage (tablets/d)	Time1 (months)	Minimum dosage (g/d)	Time2 (months)	Clinical manifestations						
									LE	UL	GD	AS	DS	IS
1	F	78	Panic disorder	10–18	3	1.25	25	B	0	0	0	++	+	+
								A	+++	++	+++	+++	++	+++
2	F	76	Sleep-associated leg spasms, liver cirrhosis,upper gastrointestinal bleeding	6–12	2.5	1.5	20	B A	0 +++	0 +	0 +	0 ++	0 +	0 ++
														
3	F	74	Cerebral infarction, poststroke depression	6–10	3	1.25	24	B A	0 +++	0 ++	0 ++	+ +++	++ +++	+ ++
														
4	F	47	Somatization disorder	8–12	2.5	1.25	13	B A	0 ++	0 0	0 +	++ +++	+ ++	0 ++
														
5	M	56	Postencephalitis	6–15	3.5	1.5	4.5	B	0	0	0	0	0	0
								A	+++	+	++	+++	++	++
														
6	F	52	Anxiety and depressive disorder	9–12	2	1.75	5	B A	0 +++	0 +	+ ++	++ +++	++ ++	++ +++
														
7	F	67	Anxiety and depressive disorder	6–9	3	1.5	31	B A	0 +++	0 0	0 +	+ ++	++ ++	+ +++
														
8	F	71	Somatization disorder	6–12	2.5	1.25	15	B A	0 +++	0 +	0 ++	++ +++	+ ++	0 +++
														
9	M	68	Cerebral infarction, poststroke depression	6–13	1.5	1.75	26	B A	0 ++	0 +	0 0	0 ++	++ ++	+ ++
														

M, male; F, female; LE, lower extremity; UL, upper limb; GD, general discomfort; 0, no symptoms; +, mild symptoms; ++, moderate symptoms; +++, severe symptoms. B, before treatment; A, after treatment. Time 1, time of onset of restless leg syndrome (RLS) after taking Madopar; Minimum dosage, minimum dose of Madopar at the onset of RLS; Time 2, time of onset of RLS to visit. Clinical manifestations, clinical manifestations before and after taking Madopar.

**Table 2:** Comparison of anxiety, depression, and insomnia scores of the nine patients.

		1	2	3	4	5	6	7	8	9	$\bar{x}$ ± *s*	*P*
HARS	B	25	4	18	25	7	22	16	23	10	16.67 ± 2.66	<0.0001
	A	52	26	50	42	38	45	27	47	26	39.22 ± 3.50	
HDRS	B	22	12	33	22	6	30	30	22	30	23.00 ± 3.03	<0.05
	A	33	25	62	31	32	33	33	32	32	34.78 ± 3.50	
ISI	B	10	7	10	5	3	17	11	5	10	8.667 ± 1.40	<0.0001
	A	26	13	19	21	17	26	25	25	19	21.22 ± 1.54	

B, before taking Madopar; A, after taking large-dose Madopar; HARS, Hamilton Anxiety Rating Scale; HDRS, 24-item Hamilton Depression Rating Scale; ISI, Insomnia Severity Index; p-value, Student’s t-test.

All nine cases were asked to gradually reduce their dose of Madopar. Low doses of long-acting dopamine agents, dopamine receptor agonists, α2δ calcium channel ligands, clonazepam, and other drug treatments were administered. All psychiatric symptoms were greatly alleviated but did not fully disappear and lasted for approximately 2 years. The severity of symptoms in seven patients with more than 6 months of disease course was significantly improved, but after 3 months of treatment, there was no obvious further improvement of symptoms and fluctuations were present.

As shown in **Table 3**, there was a significant difference in the IRLS scores 1 month before and after treatment. During the first month of follow-up, IRLS scores of all patients were significantly lower than the initial assessment [21.22 + 2.05 points (indicative of severe symptoms) compared to 35.33 ± 2.40 points; *P* < 0.0001]. At the 3-month follow-up, the IRLS scores of patients were significantly lower than the first month of follow-up (*P* = 0.001). Finally, at the 6-month follow-up, the IRLS scores were 13.89 ± 5.06 points, indicating moderate severity. Before treatment and at the 1-month follow-up, there was statistically significant difference between IRLS scores (*P* < 0.0001); when the 3-month follow-up was compared to this, although the symptoms were improved, it was not found to be statistically significant (*P* = 0.33). And, at the 12-, 18-, and 21-month follow-ups, when compared with the 6-month follow-up, two cases at the 21-month follow-up demonstrated that the IRLS score continued to decrease, reaching a mild level of severity; however, the subsequent treatment did not provide additional benefits to the remaining seven cases. The RLS symptoms showed no obvious improvements compared to the 6-month follow-up and were still classified as moderately severe symptoms. Another patient died of primary disease (cirrhosis and hemorrhage of upper digestive tract) during the 12 months of follow-up. This demonstrated that improvements were no longer obvious after 3 months, which suggested that early diagnosis and treatment might be the key factor to improving prognosis.

**Table 3:** Treatment and outcome of treatment of the nine patients and follow-up.

Case	RLS score before treatment	Length of treatment at follow-up (months)	IRLS-RS at follow-up (months)						Treatment strategy								
			1	3	6	12	18	21	MAD (tablets/d)	CLSR (tablets/d)	PSRT (mg/d)	PRE (mg/d)	EST (mg/d)	CLO (mg/d)	PAR (mg/d)	DUT (mg/d)	OLA (mg/d)
1	39	38	25	20	16	18	25	20	3	–	50	150	–	0.5	–	60	5
2	31	10	18	12	10	die	–	–	0.5	0.5	50	75–100	–	0.5	–	–	–
3	35	13	22	20	23	18	–	–	1	0.5	50	–	–	1	20	–	2.5
4	34	19	21	18	10	12	15	–	–	1	50–100	150	2	–	–	60	5
5	37	32	20	12	10	12	13	8	–	1	50	150	–	0.5	20	–	2.5
6	36	25	23	16	10	12	10	9	1	–	100	150	–	–	–	60	2.5–5
7	33	23	20	16	16	17	16	–	–	0.5	50	100	2	–	–	60	2.5
8	36	18	22	18	20	16	18	–	–	1	50	100	–	–	–	60–90	5
9	37	36	20	16	10	15	14	12	–	1	50	200	–	0.5	20–30	–	2.5

IRLS-RS, International Restless Legs Syndrome Rating Scale; MAD, Madopar; CLSRT, carbidopa and levodopa sustained-release tablets; PSRT, piribedil SR tablets; PRE, pregabalin; EST, estazolam; CLO, clonazepam; PAR, paroxetine; DUT, dutoxetine; OLA, olanzapine.

## Discussion

RLS is a common nervous system sensory dyskinesia disease, and the clinical manifestations are extreme discomfort during rest and nocturnal sleep. Symptoms can be remitted through movement of the lower extremities, which forces patients to continue to move their limbs, therefore disturbing sleep and rest. According to the etiology, RLS can be divided into two subtypes: primary and secondary. The former etiology is unclear and may be heredity, while the latter is often due to iron deficiency, pregnancy, chronic renal failure, and other causes. In the present study, we reported for the first time that nine patients who had no family history of primary or secondary RLS showed RLS-like symptoms accompanied by anxiety and depressive symptoms, as induced by high-dose Madopar.

Previous reports and case studies have suggested that certain medications may cause or exacerbate RLS. These medications include several classes of antidepressants, including tricyclic antidepressants (8) such as imipramine (9); selective serotonin or norepinephrine reuptake inhibitors (10), such as citalopram (11), escitalopram (12), fluoxetine (13), sertraline (14), paroxetine (15), trazodone (16), venlafaxine (17), dutoxetine (18), and mirtazapine (19); and neuroleptics that have significant dopaminergic blockade (20), such as olanzapine (21), risperidone (22), and quetiapine (23). In addition, antihistamines operating on the H1 receptor (24) and selected antiemetics with dopamine antagonism such as metoclopramide (25) and prochlorperazine (26) have also been associated with RLS. However, as none of the nine patients we observed took the aforementioned drugs, RLS-like symptoms caused by these drugs were excluded.

The clinical manifestations of anxiety, depression, and somatoform disorders are complex and diverse. Other than the emotional aspects, these illnesses can manifest as different forms of somatic symptoms, such as dizziness, headache, limb weakness (especially in the lower extremities), and difficulty walking, which is likely to be misdiagnosed as a primary disease. Among the nine patients in the present study, seven had varying degrees of anxiety and depression. For example, Case 5 was misdiagnosed with Parkinson’s disease and treated with Madopar due to the spasm gait of the double lower limb after encephalitis. Since the effect of treatment was not obvious, the patient increased the dosage by themselves. RLS-like symptoms and severe anxiety, insomnia, and other psychiatric symptoms occurred with an increasing amount of medication. Case 2 was diagnosed with a sleep-related leg spasm according to the predisease clinical manifestation, and then the patient was misdiagnosed with Parkinson’s disease and treated with a high dose of Madopar, which induced RLS-like symptoms and anxiety. As one of the effective drugs to treat RLS, Madopar has been widely used in clinical practice; however, the onset of RLS is very rare and its pathogenesis should be further discussed.

In 2016, single-photon emission computed tomography imaging was used to study the pathophysiological mechanisms of RLS at the Tri-Service General Hospital, National Defense Medical Center (27). The results showed a significantly reduced uptake in striatal dopamine transporter (DAT) density and activity in RLS patients (27). This study supported that symptoms of RLS resulted from the striatum due to dopaminergic system dysfunction (27).

To date, many studies have shown that using drugs such as levodopa and other dopamine agonists can significantly improve the symptoms of RLS; therefore, the central dopaminergic nervous system (particularly the nigra-striatum system or intermediate cortical system) has been considered to be associated with the onset of RLS. In the present group, patients displayed RLS symptoms after the use of long-term high doses of Madopar (from the use of Madopar to the onset of RLS-like symptoms), suggesting that the cause was the dysfunction of the central dopamine system. A number of studies have reported that the use of dopamine drugs in the treatment of RLS may promote symptom deterioration, reverse jump, and other adverse reactions. This is especially true after the long-term use of levodopa, as the proportion of deteriorated symptoms is 18–80% (28). Since these adverse reactions are more common in patients with long-term high doses of levodopa treatment, it suggests that the mechanism of symptom deterioration may be associated with dopamine overdose in the central nervous system (29). Therefore, we speculate that the mechanism of RLS-like symptoms in the nine patients of the present study may be caused by excessive dopamine in the central nervous system after the use of long-term high doses of dopaminergic agents.

High concentrations of dopamine can excite D1 receptors and cause D1 receptor-related pain, which results in periodic limb movements. Several studies have shown that certain concentrations of external toxic substances [such as levodopa, dopamine (DA)] may damage dopamine transporters (DAT), therefore significantly reducing their abundance. Additionally, compared to the mitochondria, DAT is more sensitive to injury stimulation from external toxic substances. Before the cells’ mass death, the number of DAT on the cell membranes is significantly reduced. The remaining DAT functions display compensatory hyperfunction and are therefore eliminated due to the reciprocal inhibition, which allows them to ingest more dopamine and its metabolites into the cells. This results in a large number of free radicals and the inhibition of the mitochondrial respiratory chain, and eventually causes cell death. Therefore, it is speculated that the long-term use of dopamine in patients of the present study may lead to a reduction in the number of dopamine receptors in the brain and spinal cord or a decrease in DAT function, finally resulting in dopaminergic systemic dysfunction and the occurrence of RLS symptoms.

In the present study, nine patients with long-term high doses of dopaminergic drugs displayed RLS symptoms, while the original symptoms of anxiety, depression, insomnia, and somatization appeared or were aggravated. Current epidemiological studies report that RLS is a common cause of insomnia, and the rate of comorbidity with depression and anxiety is high (30). Winkelmann et al. (31) assessed 238 RLS patients with a standardized diagnostic interview [Munich-Composite International Diagnostic Interview for DSM-IV (*Diagnostic and Statistical Manual of Mental Disorders, Fourth Edition*)]. Rates of anxiety and depressive disorders were compared between them and 2,265 community respondents from a nationally representative sample. RLS patients revealed an increased risk of having anxiety and depressive disorders with particularly strong associations with panic disorder, generalized anxiety disorder, and major depression (31). Moreover, the Baltimore Epidemiologic Catchment Area follow-up study suggested a strong association between RLS and major depressive disorder and/or panic disorder (32). An anonymous survey study in Appalachia suggested that those with RLS were significantly more likely to indicate a history of depression and anxiety and report sleep impairments both 4 and 7 days/week, with a mean sleep duration <5 h/night (33). These associations increased in both strength and magnitude with increasing symptom frequency (33). More recently, a study on the clinical characteristics of RLS in adult patients from Peking Union Medical College Hospital demonstrated that primary RLS patients suffer from poor sleep and are more susceptible to anxiety and depression (34). The scores of Hospital Anxiety and Depression Scale for depression and anxiety were significantly correlated with those of the Pittsburgh Sleep Quality Index and IRLS (34).

The underlying cause of the high incidence of RLS and anxiety and depression is unclear. It is possible that these illnesses may share a basic pathophysiological mechanism leading to their development. Pan et al. (35) explored the regional gray matter (GM) density in depressed drug-naïve RLS patients using voxel-based morphometry, which showed that GM density of the bilateral anterior cingulate cortex (ACC) was significantly reduced in RLS patients with depressive symptoms (RLS-D) compared to RLS patients without depressive symptoms or healthy controls. Additionally, a significant negative correlation between right ACC density and HDRS scores and duration of depressive symptoms in patients with RLS-D was found (35). It was speculated that depressive symptoms are associated with GM abnormalities in the ACC of patients with RLS.

In the present study, anxiety was more obvious than the depressive symptoms in our patients; however, there is no evidence to suggest its mechanism to date, thereby requiring further discussion. By administering small doses of long-acting dopamine agents, dopamine receptor agonists, α2δ calcium channel ligands, clonazepam, and other treatment to this group of patients, the IRLS score gradually declined and symptoms improved, but RLS symptoms did not completely disappear. Here, the prognosis of RLS was significantly different from secondary RLS patients, as they typically demonstrated complete disappearance of symptoms after cause elimination. It is suggested that long-term high doses of Madopar cause excessive accumulation of dopamine in the central nervous system, thereby irreversibly decreasing the number of dopamine receptors or DAT function, resulting in the persistence of clinical RLS-like symptoms.

The present study reports drug-induced (high-dose levodopa) RLS, which is different from the idiopathic RLS in treatment; however, the method treatment is identical. First, the dose of levodopa was gradually reduced, but as the clinical symptoms of the patients were severe, measures needed to be taken accordingly, depending on the patient’s condition and their accompanying anxiety, depression, and insomnia. We referenced the European guidelines on management of restless legs syndrome:report of a joint task force by the European Federation of Neurological Societies, the European Neurological Society and the European Sleep Research Society (36). According to the recommendations and precautions for drug treatment, the principle of individualization was followed, simultaneously supplemented by physical therapy (hot water bath before sleep, limb massage, etc). Thus, the clinical symptoms of the nine patients were relieved to varying degrees. The specific method recommends the minimization and withdrawal of the use of Madopar. The therapy was changed to a small dose of a long-acting dopamine agent (Xining 100–200 mg/day) to reduce the risk of dosage increase. Madopar was finally discontinued in five of the nine patients; however, Case 1 was found to have difficulty when the Madopar dose was reduced to 3 pills/day. A small dose of a dopamine receptor agonist and a small-to-moderate dose of a α2δ calcium channel ligand (pregabalin) were added depending on the severity of the patient’s symptoms. To improve the symptoms of anxiety, depression, and insomnia, the therapy was supplemented by small-dose paroxetine, duloxetine, and olanzapine; an obvious curative effect was achieved by all of them.

In summary, clinicians should be mindful of differential diagnoses when patients present with walking difficulties and limb stiffness. In addition to the original diseases, anxiety, depression, and somatization disorders should be considered. In particular, clinicians should strengthen the management of patients who use dopaminergic agents to reduce the great physical and mental adverse events due to misdiagnosis and mistreatment.

## Consent for Publication

The nine patients gave written consent for both participation and publishing the data in a scientific journal. They understood that the information will be published without their names attached, but that full anonymity cannot be guaranteed. They understood that the material may be published and placed on worldwide website and journals. Both the printed version and the website are seen and read by doctors, journalists, and members of the public. The material will not be used for advertising or packaging.

## Ethics Statement

This study was approved by the Ethics Committee of the First People Hospital of Huainan, and written informed consent was obtained from the patient for publication of this case report.

## Author Contributions

LZ, MZ, WZ: study design and critical revision of the manuscript. LZ, MZ: collection and interpretation of data. LZ: analysis data and drafting of the manuscript. JL, CR, MX, CY: collection data. All authors approved the final version for publication.

## Funding

This work was supported by the Chinese Academy of Medical Sciences Innovation Fund for Medical Sciences (2016-I2M-1-006).

## Conflict of Interest Statement

The authors declare that the research was conducted in the absence of any commercial or financial relationships that could be construed as a potential conflict of interest.
